# An Efficient Computational Model for Magnetic Pulse Forming of Thin Structures

**DOI:** 10.3390/ma14247645

**Published:** 2021-12-12

**Authors:** Mohamed Mahmoud, François Bay, Daniel Pino Muñoz

**Affiliations:** MINES Paris-Tech, PSL-Research University, CEMEF—Center for Material Forming, CNRS UMR 7635, BP 207, 1 Rue ClaudeDaunesse, CEDEX, 06904 Sophia Antipolis, France; francois.bay@mines-paristech.fr (F.B.); daniel.pino_munoz@mines-paristech.fr (D.P.M.)

**Keywords:** high-speed forming, magnetic pulse forming, computational mechanics, solid-shell finite element

## Abstract

Electromagnetic forming (EMF) is one of the most popular high-speed forming processes for sheet metals. However, modeling this process in 3D often requires huge computational time since it deals with a strongly coupled multi-physics problem. The numerical tools that are capable of modeling this process rely either on shell elements-based approaches or on full 3D elements-based approaches. The former leads to reduced computational time at the expense of the accuracy, while the latter favors accuracy over computation time. Herein, a novel approach was developed to reduce CPU time while maintaining reasonable accuracy through building upon a 3D finite element analysis toolbox which was developed in CEMEF. This toolbox was used to solve magnetic pulse forming (MPF) of thin sheets. The problem was simulated under different conditions and the results were analyzed in-depth. Innovative techniques, such as developing a termination criterion and using adaptive re-meshing, were devised to overcome the encountered problems. Moreover, a solid shell element was implemented and tested for thin structure problems and its applicability was verified. The results of this element type were comparable to the results of the standard tetrahedral MINI element but with reduced simulation time.

## 1. Introduction

Lightweight structures have many applications, especially in aerospace and automotive industries. The huge demand on lightweight structures requires improvements in the forming processes that would lead to cost reduction while maintaining structural resistance. High-speed forming processes, such as explosive forming discussed by Mynors and Zhang [1], electro-hydraulic forming (EHF) by Rohatgi et al. [2] and electromagnetic forming (EMF) by Zittel [3], have been proposed in the literature to overcome the disadvantages of the conventional forming methods. The study of these problems has been cumbersome for a long period of time. However, studying such problems has become more feasible with the advancements in the computing power and the progress in computational simulations.

Multi-physics simulation is a wide domain that contains various problems, including fluid-structure [4], thermal-structural and electromagnetic-structure interactions [5]. This work is mainly concerned with electromagnetic-structure simulations. Nevertheless, there are two types of this simulation. First, the simulation related to using materials having unique electric-magnetic properties, such as functionally graded piezoelectric material (FGPM) and piezomagnetic (PM) materials [6]. Second, the problems that include external magnetic sources that induce magnetic forces causing the metallic structure to deform, known as electromagnetic forming (EMF) [7].

The electromagnetic forming (EMF) process applies an intense electromagnetic pulse on a metallic component, inducing plastic deformations. It is a high-speed forming process that has strain rates ranging from $10^{3}\;s^{-1}$ to $10^{4}\;s^{-1}$. Psyk et al. [8] discussed, in detail, the advantages of high-speed forming processes over the conventional forming processes. First, there is a lack of contact between the tool and the workpiece, and thus no lubrication is needed. Second, there is improved formability with respect to the conventional forming processes. In this process, an intense magnetic field generated by a coil is applied on an adjacent electrical conductive workpiece. The induced current along with the applied magnetic field produce Lorentz body forces on the workpiece. These forces supply additional momentum and energy to the workpiece, causing deformations [9]. Unger et al. [10] stated that the electromagnetic part of the system is highly dependent on the spatio-temporal evolution of the deformation of the workpiece. Therefore, designing this process remains cumbersome as it deals with strongly coupled multi-physics phenomena.

Recently, many endeavors have been made to simulate this problem. Most of these approaches were mainly restricted to axisymmetric geometries or small deformation problems. Fenton and Daehn [11] tackled the simulation problem of magnetic pulse welding and Imbert et al. [12] worked on the simulation of a corner fill operation process, though both of them worked on axisymmetric geometries. Moreover, Schinnerl et al. [13] tackled the simulation of 3D magnetic pulse forming but in small deformations. However, three-dimensional modeling along with plastic deformations are necessary to simulate a real world metal forming process using electromagnetic forming. The numerical simulation of this problem requires high standards of finite element formulation since the workpieces exhibit large bending and plasticity. Therefore, low order elements are not favorable in such applications as they exhibit a locking effect in bending problems which deteriorates the accuracy of the results. There are many solutions to overcome the locking effect. One possibility is to work with higher order elements [14]. Nonetheless, these formulations require complicated meshing procedures and complicated handling of the contact algorithms. Another solution is to use the method of compatible modes or enhanced assumed strains [15,16]. However, these approaches require more internal element variables to be determined, which leads to a higher computational cost. Recently, Belytschko and Bindeman [17], Liu et al. [18] and Reese et al. [19] have proposed a methodology to combine the assumed enhanced strain method with the reduced integration technique and hourglass stabilization. This approach showed robust deformation behavior along with low numerical cost. A new category of elements are based on the same concept, called solid shell elements [20,21]. Unger et al. [10] developed a model for 3D simulation of electromagnetic forming using a solid shell element [22] for the mechanical solution to reduce the computational cost. However, Unger et al. [10] addressed only the simple problem of a rectangular shape workpiece, which did not represent a real life application problem.

Additionally, many multi-physics commercial software already exist in the market, such as LS-DYNA [23]. The electromagnetic computations in LS-DYNA are based on the coupling between finite elements for solid bodies and boundary elements [24] for the surrounding air [25]; therefore, the air domain is not meshed. This can be more adapted for moving bodies but can significantly increase the computational time due to the fact that the boundary element system leads to solving a small linear system but with a full matrix. On the other hand, this work is complementary to the work of Alves Z and Bay [7], in which the authors use a tetrahedral solid element, called the Nedelec element [26], in which all fields are defined on the solid element’s edges instead of its nodes. Thus, all bodies in the system are different, solely by the material parameter, and have the same finite element. This approach can be quite fast since it deals with a single element type in the system. Moreover, since the approach produces a single linear system for the resolution of the magnetic potential, the parallelism is also simplified [27]. Finally, remeshing is more adapted to this approach since tetrahedra are the most suited elements for enabling automatic and adaptive remeshing. It is one of the main advantages of our tool and the work carried out here is aimed at developing an original strategy in order to preserve the efficiency of this strategy.

The main novelty of this work is the study of an application of the EMF processes, which is the magnetic pulse forming (MPF) of thin sheets. This process can be used in either direct forming, used with highly conductive materials, or indirect forming, used with less conductive materials. Although the magnetic pulse forming problem can be simulated using axisymmetric model [28], we have chosen to use a 3D approach to model the problem for some reasons. First, the approaches developed in this paper are generic and are used to solve general 3D problems in the future. Second, the ultimate goal is to include the effect of plastic anisotropy in the future simulations and implementing 3D constitutive equations that are more convenient with most of the anisotropic yield criteria. Herein, the simulations are carried out using different element types: MINI element [29] and solid shell element [20]. This solid shell element was developed and modified to fit magnetic pulse forming applications [30]. Using the solid shell element instead of the MINI element reduces the computational cost of the simulation dramatically.

The paper is divided as follows: Section 2 discusses the modeling of the electromagnetic problem and the mechanical problem, in brief, along with a glimpse of the implementation strategy adopted for these simulations. Section 3 tackles the description of the magnetic pulse forming application, its finite element description, the results and their physical interpretation. Section 4 is an extensive study of the difficulties that have been encountered in the problem and some proposed solutions to overcome these difficulties. Finally, Section 5 states the concluding remarks.

## 2. Modeling of the Magnetic Pulse Forming Process

Multi-physics simulations, including electromagnetic simulations, can be very computationally expensive. Additionally, design processes that include multiple simulation iterations and optimization processes require high computational power to be carried out. Thus, it is important to select the most appropriate numerical method to solve these problems in a reasonable time while maintaining good accuracy.

A numerical toolbox based on finite elements methods for the electromagnetic forming applications has been developed to solve electromagnetic forming problems [31,32]. This toolbox is a coupling between FORGE—for the mechanical modeling of large deformation—and MATELEC—which solves the electromagnetic wave propagation problem—based on the Maxwell’s equations. The following subsections explain the electromagnetic and mechanical models used in the simulation of the magnetic pulse forming process.

### 2.1. Electromagnetic Model

#### 2.1.1. Maxwell’S Equations and the Potential Formulation

The electromagnetic solver is based on the well-known Maxwell’s electromagnetic field equations:

(1) $$\vec{\nabla }\times \vec{E}=-\frac{\partial \vec{B}}{\partial t}$$

(2) $$\vec{\nabla }\times \vec{H}=\vec{J}$$

(3) $$\vec{\nabla }\cdot \vec{D}=0$$

(4) $$\vec{\nabla }\cdot \vec{B}=0$$

where
$\vec{E}:$ Electric field intensity$\vec{D}:$ Electric flux intensity$\vec{H}:$ Magnetic field intensity$\vec{B}:$ Magnetic flux intensity$\rho^{e}:$ Electric charge density$\vec{J}:$ Electric current density

- Equation (1): (Maxwell Faraday) represents the electric induction due to a varying magnetic field.
- Equation (2): (Maxwell Ampere) represents the creation of a magnetic field due to a passing electric current.
- Equation (3): (Maxwell gauss) represents the conservation of electric charge in the material.
- Equation (4): Represents the conservation of the induced magnetic field in the material.

Although, a reduced form of Maxwell-gauss Equation (3) is used in which $\rho^{e}=0$, since there is no fixed electric charge to be considered in this problem. Moreover, a reduced form of Maxwell-Ampere Equation (2) is used in the current applications of metal forming, since electromagnetic wave propagation may be neglected [33]; thus, ($\frac{\partial \vec{D}}{\partial t}=0$).

Then, the model is completed with the constitutive relations:

(5) $$\vec{B}=\mu_{H}\vec{H};\vec{J}=\frac{1}{\rho_{E}}\vec{E}$$

where $\mu_{H}$ is magnetic permeability and $\rho_{E}$ the electrical resistivity. These material parameters depend on the temperature and $\mu_{H}$ depends also on the intensity of the magnetic field $\left|\right|\vec{H}\left|\right|$.

In many cases, it is more convenient to express this system of equations in potential formulation ($\vec{A}$,ϕ) [34] where $\vec{A}$ is the vector potential function and ϕ is the scalar potential function that can be represented by the following equations:

(6) $$\vec{\nabla }\cdot \vec{B}=0\Rightarrow \vec{B}=\vec{\nabla }\times \vec{A}$$

Combining Equation (1) with Equation (6):

(7) $$\begin{array}{cc} \vec{\nabla }\times \vec{E}=-\frac{\partial \vec{B}}{\partial t} & \Rightarrow \vec{\nabla }\times \vec{E}=-\frac{\partial }{\partial t}\left(\vec{\nabla }\times \vec{A}\right)\\ \; & \Rightarrow \vec{\nabla }\times \left(\vec{E}+\frac{\partial \vec{A}}{\partial t}\right)=0 \end{array}$$

Since for any scalar function ϕ, $\vec{\nabla }\times \left(-\vec{\nabla }\phi \right)=0$ holds, then

(8) $$\begin{array}{cc} \; & \Rightarrow \vec{E}+\frac{\partial \vec{A}}{\partial t}=-\vec{\nabla }\phi \\ \; & \Rightarrow \vec{E}=-\vec{\nabla }\phi -\frac{\partial \vec{A}}{\partial t} \end{array}$$

Finally, after substitution of ($\vec{A},\phi$) in Maxwell’s equation and considering law of the charge conservation, the final equations can be written as follows:

(9) $$\begin{array}{cc} \frac{1}{\rho_{E}}\frac{\partial \vec{A}}{\partial t}+\vec{\nabla }\times \left(\frac{1}{\mu_{H}}\vec{\nabla }\times \left(\vec{A}\right)\right) & =-\frac{1}{\rho_{E}}\vec{\nabla }\left(\phi \right)\\ \vec{\nabla }\cdot \left(\frac{1}{\rho_{E}}\vec{\nabla }\phi \right)+\vec{\nabla }\cdot \left(\frac{1}{\rho_{E}}\partial_{t}\vec{A}\right) & =0 \end{array}$$

This is a four variables ($\phi ,A_{x},A_{y},A_{z}$) four equations system instead of six variables for a double vector field formulation. Equation (9) is discretized in space by Nedelec elements [26] and $\vec{A}$ is solved at the edges while ϕ is solved at the nodes.

#### 2.1.2. Weak Formulation and Discretization of Electromagnetic Problem

**Figure 1:**
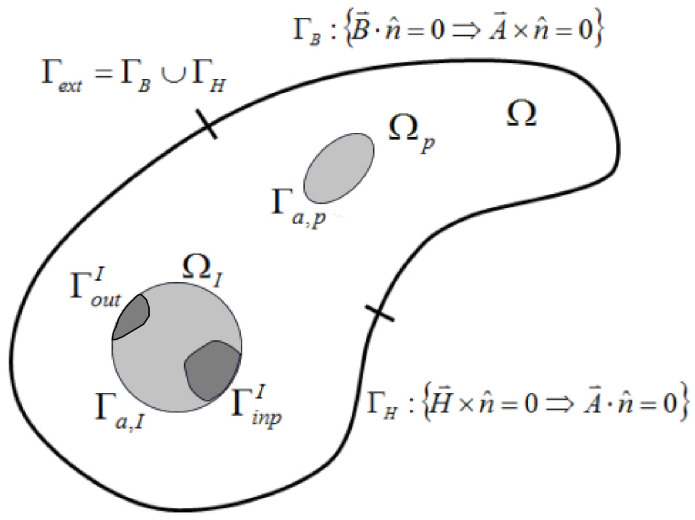
Boundaries of an EMF process. Ω represents the global domain solids + surroundings. $\Omega_{P}$ is the workpiece. $\Omega_{I}$ represents the inductor domain. The electrical input and output connections of the inductor are given by $\Gamma_{inp}^{I}$ and $\Gamma_{out}^{I}$ [35].

The electromagnetic problem consists of a single domain, as indicated in Figure 1. The domain (Ω) is subdivided into three subdomains: the coil or inductor ($\Omega_{I}$), the workpiece ($\Omega_{P}$) and the surrounding air ($\Omega_{a}$)

Reese et al. [28] utilized Coloumb gauge condition in order to guarantee uniqueness of solution. This condition indicates that:

(10) $$\vec{\nabla }\cdot \vec{A}=0$$

Hence, the second equation of Equation (9) is reduced to:

(11) $$\vec{\nabla }\cdot \left(\frac{1}{\rho_{E}}\vec{\nabla }\phi \right)=0$$

Therefore, the weak form of the electromagnetic differential equations in Equation (9) will take the following form:

(12) $$\begin{array}{c} \langle \vec{\Psi },\frac{1}{\rho_{E}}\partial_{t}\vec{A}+\vec{\nabla }\times \frac{1}{\mu_{H}}\vec{\nabla }\times \vec{A}+\frac{1}{\rho_{E}}\vec{\nabla }\phi \rangle =0\\ \langle \varphi ,\vec{\nabla }\cdot \left(\frac{1}{\rho_{E}}\vec{\nabla }\phi \right)\rangle =0 \end{array}$$

$\;\mathrm{for}\;\mathrm{all}\;\vec{\Psi }\in H^{\mathrm{curl}\;}\;\mathrm{and}\;\varphi \in H_{0}^{1}$. Where,

- Space of functions vanishing at the boundary $H_{0}^{1}\left(\Omega \right)\subset H^{1}\left(\Omega \right)$.
  (13) $$H_{0}^{1}\left(\Omega \right)=\left\{\varphi \in H^{1}\left(\Omega \right)/\varphi =0\in \partial \Omega \right\}$$
- Space of vector functions with square-integrable curl.
  (14) $$H^{\mathrm{curl}\;}\left(\Omega \right)=\left\{\vec{\Psi }\in {\left(L^{2}\left(\Omega \right)\right)}^{3}/\vec{\nabla }\times \vec{\Psi }\in {\left(L^{2}\left(\Omega \right)\right)}^{3}\right\}$$
- Inner products: The following notation for the inner product of the spaces will allow simplifying the notation for the weak forms.
  (15) $$\int_{\Omega }f\cdot gd\Omega =\langle f,g\rangle$$

Alves Zapata [27] developed the detailed mathematical model and considered natural conditions to reach the final weak form:

(16) $$\begin{array}{c} \langle \vec{\Psi },\sigma \partial_{t}\vec{A}\rangle +\langle \vec{\nabla }\times \vec{\Psi },\frac{1}{\mu }\vec{\nabla }\times \vec{A}\rangle +\langle \vec{\Psi },\frac{1}{\rho_{E}}\vec{\nabla }\phi \rangle =0\\ \langle \vec{\nabla }\varphi ,\frac{1}{\rho_{E}}\vec{\nabla }\phi \rangle =0 \end{array}$$

Afterwards, the approximate fields solutions representing the finite elements discretization is defined as:

(17) $$\begin{array}{cc} \phi (t,\vec{x}) & \approx \phi^{h}\left(t,\vec{x}\right)=\sum_{n}\phi_{n}\left(t\right)\varphi_{n}\left(\vec{x}\right)\\ \vec{A}\left(t,\vec{x}\right) & \approx {\vec{A}}^{h}\left(t,\vec{x}\right)=\sum_{d}a_{d}\left(t\right){\vec{\Psi }}_{d}\left(\vec{x}\right) \end{array}$$

where $\varphi_{n}\left(\vec{x}\right)$ are the nodal shape functions and ${\vec{\Psi }}_{d}\left(\vec{x}\right)$ are the edge shape functions (Nedelec elements). Alves Zapata [27] addresses in more details the interpolation functions and finite element formulation of this problem.

Finally, Lorentz forces can be computed from the potential formulation as follows:

(18) $$\begin{array}{cc} {\vec{F}}_{lorentz} & =\vec{J}\times \vec{B}\\ {\vec{F}}_{lorentz} & =\frac{1}{\rho_{E}}\left[-\frac{\partial \vec{A}}{\partial t}\times \left(\vec{\nabla }\times \vec{A}\right)\right] \end{array}$$

Lorentz force is only function in $\vec{A}$, which can be computed directly after solving for $\vec{A}$.

### 2.2. Solid Mechanics Model

The second part of the simulation is related to solid mechanics simulation in which the electromagnetic forces are transferred to the metal part, causing deformation. In this paper, two different formulations are considered in the simulation results: a mixed pressure-velocity element formulation (MINI) element [29] and an enhanced assumed strain element formulation [20]. In the following part, we will present a glimpse of both formulations.

#### 2.2.1. Mini Element Formulation

**Figure 2:**
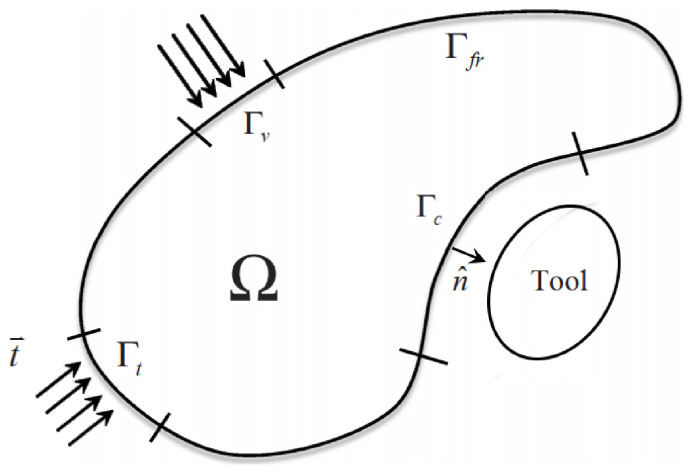
Representation of the domain Ω and the boundary conditions [27].

For the MINI element, the strong form of the mechanical problem is defined by conservation equation along with the boundary conditions. Considering the decomposition of the stress tensor into spherical and deviatoric parts, the mechanical problem is represented on the domain Ω and the external boundary Γ=∂Ω, shown in Figure 2. The boundary is decomposed to several parts depending on the type of loading applied: $\Gamma =\Gamma_{fr}\cup \Gamma_{t}\cup \Gamma_{v}\cup \Gamma_{c}$ where:

- $\Gamma_{fr}$: Free surface boundary.
- $\Gamma_{t}$: Imposed external traction boundary.
- $\Gamma_{v}$: Imposed external velocity boundary.
- $\Gamma_{c}$: Contact condition on the boundary with other tools (rigid or deformable).

The system of equations representing the mechanical problem can be summarized as follows:

(19) $$\left\{\begin{array}{c} \vec{\nabla }\cdot s-\vec{\nabla }p={\vec{f}}^{ext}\\ \vec{\nabla }\cdot \vec{v}=-\left(\frac{\dot{p}}{\kappa }\right)\\ \vec{v}={\vec{v}}_{0}\;\mathrm{on}\;\partial \Omega_{v}\\ \vec{t}={\vec{t}}_{0}\;\mathrm{on}\;\partial \Omega_{t} \end{array}\right.$$

The first two equations represent the conservation of momentum in the system. Although, the representation used here is divided to deviatoric stress and pressure that will help later in developing the mixed formulation element approach.

Third equation represents Dirichlet boundary condition and fourth equation represents Neumann boundary condition.

The weak form is based on a mixed velocity-pressure formulation. The formulation is written for test functions ($v^{*}$,$p^{*}$) as follows:

(20) $$\left\{\begin{array}{c} \int_{\Omega }s\left(\vec{v}\right):\dot{\varepsilon }\left({\vec{v}}^{*}\right)d\Omega -\int_{\Omega }p\vec{\nabla }\cdot {\vec{v}}^{*}d\Omega -\int_{\Omega }{\vec{F}}_{lorentz}\cdot {\vec{v}}^{*}d\Omega -\int_{\partial \Omega_{t}}{\vec{t}}_{0}\cdot {\vec{v}}^{*}d\Gamma =0\\ \int_{\Omega }p^{*}\left(-\vec{\nabla }\cdot \vec{v}-\frac{\dot{p}}{\kappa }\right)d\Omega =0\\ \forall \left({\vec{v}}^{*},p^{*}\right)\in V^{0}\times P \end{array}\right.$$

where $s(\vec{v})$ is the deviatoric stress, p is the pressure, $\vec{v}$ is the velocity vector, $\dot{\varepsilon }$ the strain rate and κ is the bulk modulus.

(21) $$\left\{\begin{array}{c} V^{0}=\left\{{\vec{v}}^{*}\in {\left(H^{1}\left(\Omega \right)\right)}^{3},{\left.{\vec{v}}^{*}\right|}_{\partial \Omega_{v}}=\vec{0}\mathrm{sur}\Omega \right\}\\ P=L^{2}\left(\Omega \right) \end{array}\right.$$

$H^{1}$ is the Sobolov space and $L^{2}$ is the $L^{p}$ space of square functions summed on Ω.

This problem has a unique solution. Although, from a numerical point of view, numerical instabilities can arise depending on the choice of the discretization space for *v* and *P*. In order to ensure the stability of this approach, the numerical formulation should pass the Brezzi condition [36]. The P1+/P1 discretization allows to pass Brezzi condition leading to a well posed discrete problem.

**Figure 3:**
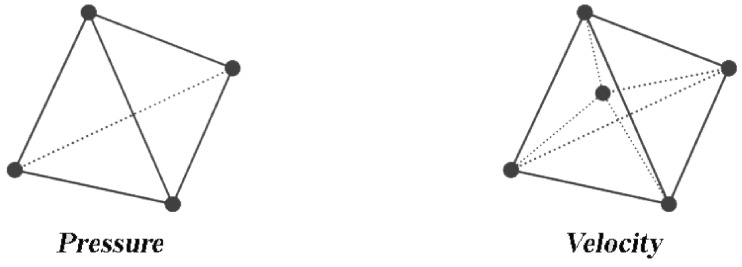
Degrees of freedom for the velocity and pressure for the tetrahedral element P1+/P1.

The element used for this formulation is tetrahedral linear 3D element in which both the pressure and velocity are linearly interpolated. However, the velocity interpolation is enhanced by a dot at the center of the element, called “bubble”, as shown in Figure 3.

The following equation presents the interpolation function for both the velocity and pressure. The velocity field is divided into two parts: linear and bubble interpolation functions.

(22) $$\left\{\begin{array}{c} {\vec{v}}_{h}\left(\vec{x}\right)=\sum_{k=1}^{Nbnode}N_{k}^{l}\left(\vec{x}\right){\vec{V}}_{k}^{l}+\sum_{j=1}^{Nbelt}N_{j}^{b}\left(\vec{x}\right){\vec{V}}_{j}^{b}\\ p_{h}\left(\vec{x}\right)=\sum_{k=1}^{Nbnode}N_{k}^{l}\left(\vec{x}\right)P_{k} \end{array}\right.$$

where $N_{k}^{l}$, k=1⋯Nbnode are the shape function of the linear interpolation for the velocity and pressure, while $N_{j}^{b}$, j=1⋯Nbelt is the bubble function [37].

#### 2.2.2. Shb Element Formulation

On the other hand, the general variational principle of SHB element is considered for the mechanical problem from Hu–Washizu variational principle:

(23) $$\delta \pi \left(\vec{v},\dot{\tilde{\varepsilon }},\tilde{\sigma }\right)=\int_{\Omega_{e}}\delta {\dot{\varepsilon }}^{T}\cdot \sigma d\Omega +\delta \int_{\Omega_{e}}{\tilde{\sigma }}^{T}\cdot \left(\nabla_{s}\left(\vec{v}\right)-\dot{\tilde{\varepsilon }}\right)d\Omega -\delta v^{T}\cdot {\vec{f}}^{ext}=0$$

where δ denotes a variation, $\vec{v}$ the velocity field, $\dot{\tilde{\varepsilon }}$ the assumed strain rate, $\tilde{\sigma }$ the interpolated stress, σ the stress field evaluated by constitutive model, $\vec{v}$ the nodal velocities, ${\vec{f}}^{ext}$ the external nodal forces (includes Lorentz forces and other external forces in the problem) and $\nabla_{s}\left(\vec{v}\right)$ the symmetric part of the velocity gradient.

Then a simplified form of this principle is achieved by considering the interpolated stress orthogonal to the difference between the symmetric part of the velocity gradient and the assumed strain rate, which gives:

(24) $$\delta \pi \left(\dot{\tilde{\varepsilon }}\right)=\int_{\Omega_{e}}\delta {\dot{\varepsilon }}^{T}\cdot \sigma d\Omega -\delta v^{T}\cdot {\vec{f}}^{ext}=0$$

In order to avoid a locking problem, a finite element is constructed based on the enhanced assumed strain method and using reduced integration [20].

**Figure 4:**
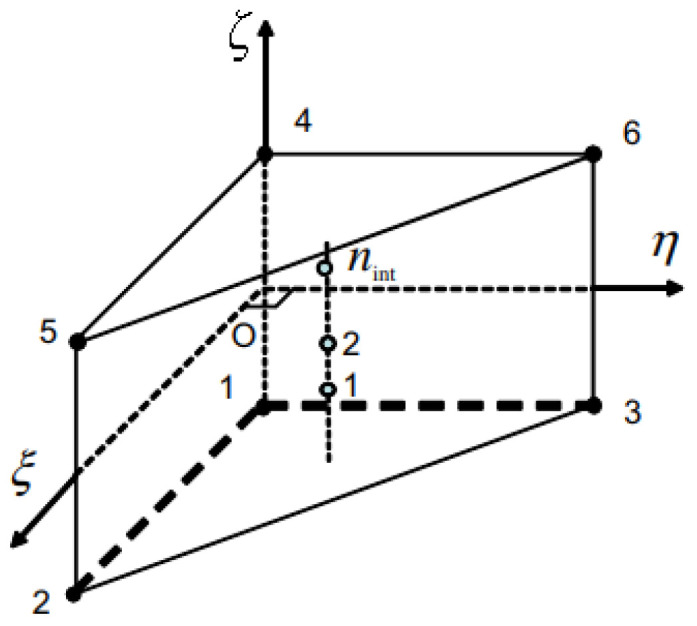
SHB element shape and integration points locations.

The element type is different from the one used for the MINI element. It is a linear prism element that interpolates the velocities as the only degrees of freedom. Figure 4 shows the element used for this formulation and the alignment of the integration point along the thickness direction ζ. Trinh et al. [20] discuss, in detail, the element interpolation along with the position of the integration points.

The interpolation of the coordinates $x_{i}$ and the displacements $u_{i}$ are as follows:

(25) $$x_{i}=x_{iI}N_{I}\left(\xi ,\eta ,\zeta \right)=\sum_{I=1}^{n}x_{iI}N_{I}\left(\xi ,\eta ,\zeta \right)$$

(26) $$v_{i}=v_{iI}N_{I}\left(\xi ,\eta ,\zeta \right)=\sum_{I=1}^{n}v_{iI}N_{I}\left(\xi ,\eta ,\zeta \right)$$

where $N_{I}$, $v_{iI}$ and $x_{iI}$ are the shape functions, the nodal velocities and the nodal coordinates, respectively. Moreover, the lowercase subscript *i* varies from 1 to 3, representing the spatial coordinates, and the uppercase subscript *I* varies from 1 to n, representing the number of nodes per element [20].

The implementation of the electromagnetic and mechanical solvers is not easy, especially the coupling between them. The following subsection gives a glimpse of the numerical implementation adopted in this work.

### 2.3. Numerical Implementation

#### 2.3.1. Coupling Algorithm

**Figure 5:**
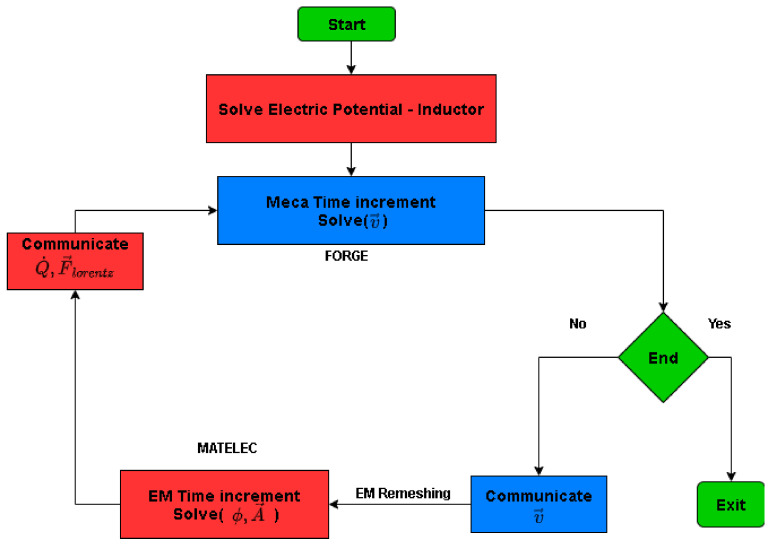
Schematic view of the coupling strategy between mechanical and electromagnetic solvers [7].

Figure 5 shows a schematic view of the coupling strategy between the electromagnetic solver and mechanical solver used to solve the MPF problem. A weak coupling is used for the electromagnetic and mechanical problems. Therefore, each solver (MATELEC, FORGE) solves its own physical problem separately, independently of the other solver. Although, after every time increment, the two solvers communicate the corresponding data and variables between each other. This is known as a loosely-coupled scheme [7]. Moreover, this approach allows adapting the mesh separately for each solver which is crucial, especially in the electromagnetic solver in which the air surrounding the moving parts should be remeshed.

#### 2.3.2. Shb Element Implementation Algorithm

The SHB element formulation uses prism elements that have been implemented in a tetrahedral element finite element software. Mahmoud et al. [30] developed a prism division algorithm that ensures that all prism elements are resembled as a set of tetrahedral elements in the software, with minimal changes in the code structure. The main idea was to divide one prism element into six overlapping tetrahedral elements. The overlapping of elements is crucial so that all the components of the original SHB stiffness matrix could be presented in at least one of the generated tetrahedral elements.

**Figure 6:**
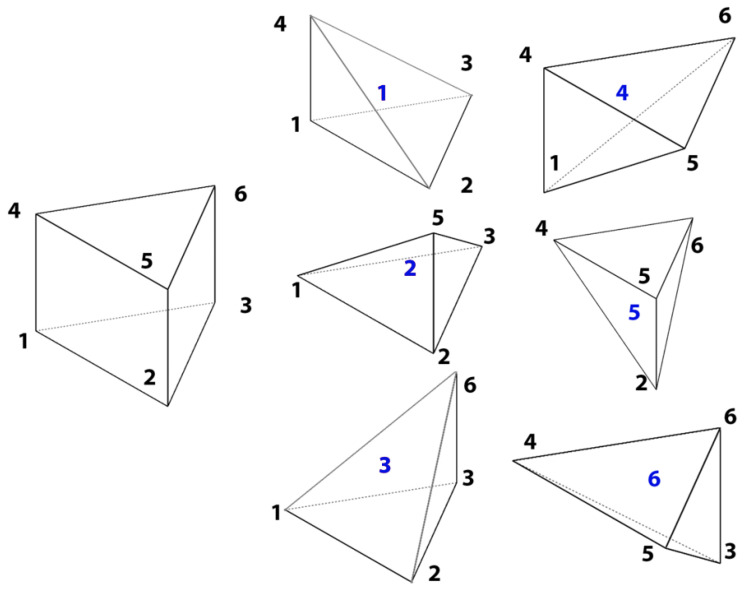
Prism division to six overlapping tetrahedral elements [30].

Figure 6 shows the corresponding tetrahedral elements generated from the divided prism element. Considering the number of tetra elements sharing each component (node) of the prism stiffness matrix, the new stiffness matrices for the tetra elements can be constructed as follows:

(27) $$element1:\left[\begin{array}{cccc} \frac{K_{11}}{4} & \frac{K_{12}}{3} & \frac{K_{13}}{3} & \frac{K_{14}}{2}\\ & \frac{K_{22}}{4} & \frac{K_{23}}{3} & \frac{K_{24}}{2}\\ & \; & \frac{K_{33}}{4} & \frac{K_{34}}{2}\\ & \; & & \frac{K_{44}}{4} \end{array}\right]element2:\left[\begin{array}{cccc} \frac{K_{11}}{4} & \frac{K_{12}}{3} & \frac{K_{13}}{3} & \frac{K_{15}}{2}\\ & \frac{K_{22}}{4} & \frac{K_{23}}{3} & \frac{K_{25}}{2}\\ & \; & \frac{K_{33}}{4} & \frac{K_{35}}{2}\\ & \; & & \frac{K_{55}}{4} \end{array}\right]$$

(28) $$element3:\left[\begin{array}{cccc} \frac{K_{11}}{4} & \frac{K_{12}}{3} & \frac{K_{13}}{3} & \frac{K_{16}}{2}\\ & \frac{K_{22}}{4} & \frac{K_{23}}{3} & \frac{K_{26}}{2}\\ & \; & \frac{K_{33}}{4} & \frac{K_{36}}{2}\\ & \; & & \frac{K_{66}}{4} \end{array}\right]element4:\left[\begin{array}{cccc} \frac{K_{11}}{4} & \frac{K_{14}}{2} & \frac{K_{15}}{2} & \frac{K_{16}}{2}\\ & \frac{K_{44}}{4} & \frac{K_{45}}{3} & \frac{K_{46}}{3}\\ & \; & \frac{K_{55}}{4} & \frac{K_{56}}{3}\\ & \; & & \frac{K_{66}}{4} \end{array}\right]$$

(29) $$element5:\left[\begin{array}{cccc} \frac{K_{22}}{4} & \frac{K_{24}}{2} & \frac{K_{25}}{2} & \frac{K_{26}}{2}\\ \; & \frac{K_{44}}{4} & \frac{K_{45}}{3} & \frac{K_{46}}{3}\\ \; & \; & \frac{K_{55}}{4} & \frac{K_{56}}{3}\\ \; & \; & & \frac{K_{66}}{4} \end{array}\right]element6:\left[\begin{array}{cccc} \frac{K_{33}}{4} & \frac{K_{34}}{2} & \frac{K_{35}}{2} & \frac{K_{36}}{2}\\ & \frac{K_{44}}{4} & \frac{K_{45}}{3} & \frac{K_{46}}{3}\\ & \; & \frac{K_{55}}{4} & \frac{K_{56}}{3}\\ & \; & & \frac{K_{66}}{4} \end{array}\right]$$

In this way, the prism element was presented implicitly through the usage of tetrahedral elements and there was no need to profoundly change the code structure used for the simulation.

## 3. Magnetic Pulse Forming Case Study

**Figure 7:**
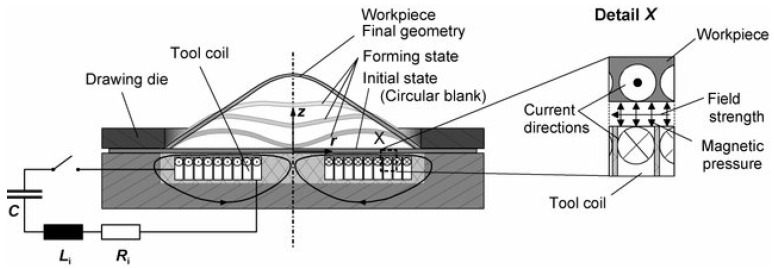
Illustration of magnetic pulse forming setup [38].

Figure 7 shows the schematic view of the free bulging process. A round flat coil is used along with a ring-shaped matrix that blocks the displacement at the circumference of the workpiece. This problem is very convenient for clarifying the coupling between the electromagnetic and mechanical solvers discussed earlier. The following subsections will tackle the details of the simulation setup with respect to the electromagnetic simulation and the mechanical simulation.

### 3.1. Electromagnetic Simulation Setup

**Figure 8:**
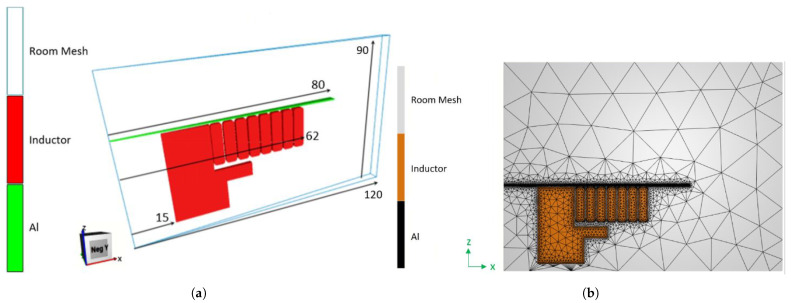
Electromagnetic simulation setup. (**a**) Geometry and dimensions of the electromagnetic simulation; (**b**) mesh configuration of the electromagnetic simulation.

Geometry: Figure 8a shows the geometry of the electromagnetic simulation with the dimensions. The aluminum workpiece has a 0.5 mm thickness and it is located 0.5 mm above the coil.

Mesh: Figure 8b shows the mesh used in the electromagnetic simulation. Three-dimensional global mesh is composed of 43,000 nodes with 260,000 tetrahedral elements. The element size is selected so that the mesh is fine in regions where strong gradients are expected and in the proximity of the metallic sheet.

Simulation properties: Table 1 shows the parameters that define the magnetic properties of the materials according to Equation (5). Additionally, it shows the parameters of the machine that produces the electromagnetic field used for the MPF process.

### 3.2. Mechanical Simulation Setup

**Figure 9:**
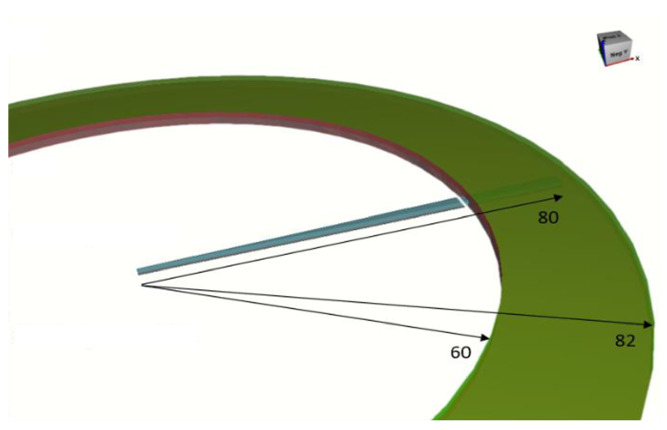
Geometry and dimensions of the mechanical simulation.

Geometry: Figure 9 shows the geometry of the $2^{\circ }$ section of the workpiece. The workpiece is fixed in the region of the green ring shown in the figure. The fixed boundary conditions come from the fact that the perimeter of the workpiece is held by a cylindrical clamp that prevent it from moving.

Mesh: Different mesh sizes and element types have been used to simulate the mechanical problem and the results were compared. In the results section, each curve will present the element type and the number of elements used for these results. Mesh study has been carried out to investigate the difference in results using different mesh sizes in Section 4.3 and the most appropriate mesh sizes are used in the results sections.

Material properties: Material properties of Al (Al1050 [39]) and Steel (AISI 4130 [40]) used in the simulation are shown in Table 2. On the other hand, Johnson–Cook law was used to describe the elastoviscoplastic behavior of the materials. Equation (30) shows the constitutive law used and Table 2 shows the corresponding constants.

(30) $$\sigma_{Y}=\left(A+B{\bar{\epsilon }}_{pl}^{n}\right)\left(1+\mathrm{error}\left(\frac{{\dot{\epsilon }}_{pl}}{{\dot{\epsilon }}_{0}}\right)\right)$$

where $\sigma_{Y}$ is the yield stress, ${\bar{\epsilon }}_{pl}$ is equivalent plastic strain, ${\dot{\epsilon }}_{pl}$ plastic strain rate and ${\dot{\epsilon }}_{0}$ initial plastic strain rate.

Boundary conditions: In the direct forming, the Aluminum workpiece is the only part in the mechanical solver. Thus, there is no contact condition added in this simulation. However, there is a bilateral sticking condition between the green ring manipulator shown in Figure 9 and the workpiece. This induces fixed boundary conditions on the circumference of the part. On the other hand, the indirect forming contains two metal parts: Aluminum and Steel. There is a sliding contact condition between Aluminum and Steel so that the friction does not affect the final deformation results. Table 3 summarizes all the boundary conditions adopted in this simulation.

### 3.3. Results Overview

This section is fully dedicated to discussing the results of the MPF problem in the utmost details possible. The following subsections will tackle two basic types of the MPF process: direct forming and indirect forming. The former, a 160 mm diameter disc-shaped workpiece, is formed by MPF directly, whereas the latter, an Aluminum disc, is placed between the coil and the Steel workpiece since Al has a much higher electrical conductivity than Steel and will help better form the Steel material [7]. Many tests have been carried out either by direct forming or indirect forming.

#### 3.3.1. Direct Forming

**Figure 10:**
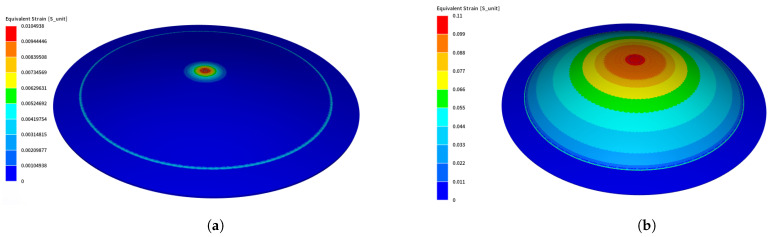
Equivalent strain of Steel and Al separately after direct forming using MINI element. (**a**) Equivalent strain of Steel at 7 kV; (**b**) equivalent strain of Al at 3 kV.

In this subsection, the results of the direct forming process are presented. Two different workpieces were tested under direct forming: 0.5 mm thick Al and 1 mm thick Steel. Equivalent strains of both Steel and Al at 7 kV and 3 kV are shown in Figure 10a,b, respectively. The equivalent strain gives some insights on the local deformation of the workpiece. It is obvious that the strain distribution on the Aluminum part is more homogeneous than in the Steel case. This is justified by the fact that Aluminum is a much better electrical conductor than Steel, which enhances the generated Lorentz forces causing distributed deformation, whereas in Steel, the deformation is concentrated at the center as it is the nearest point to the center of the coil.

**Figure 11:**
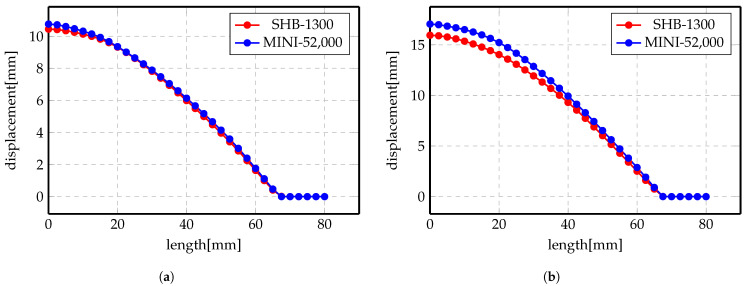
Displacement profile of direct forming process of Al. (**a**) 2 kV; (**b**) 3 kV.

**Figure 12:**
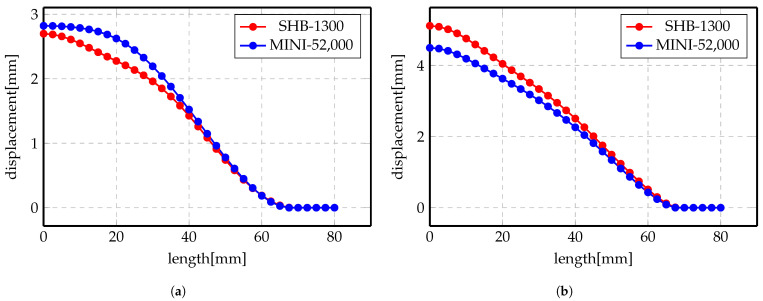
Displacement profile of direct forming process of Steel. (**a**) 5 kV; (**b**) 7 kV.

More investigations have been conducted on the deformed shapes. Figure 11a,b shows the deformation profile along the radius of the workpiece at 2 kV and 3 kV, respectively, for Al material. The simulation was conducted using two elements, MINI and SHB. Numerical results show good agreement with each other, even though the number of elements of the SHB element is much smaller than the number of the MINI elements. Likewise, the results of Steel material are presented in Figure 12a,b for 5 kV and 7 kV, respectively. There is an overall agreement among the numerical results for both of the elements.

The final deformation of the Steel material is very small and cannot be shaped through direct forming due to insufficient electrical conductivity and, consequently, not enough deformation range. Therefore, the rest of the work is focused on indirect forming in order to obtain more deformation with the help of an aluminum hammer, which is the same workpiece used in direct forming (0.5 mm thick).

#### 3.3.2. Indirect Forming

**Figure 13:**
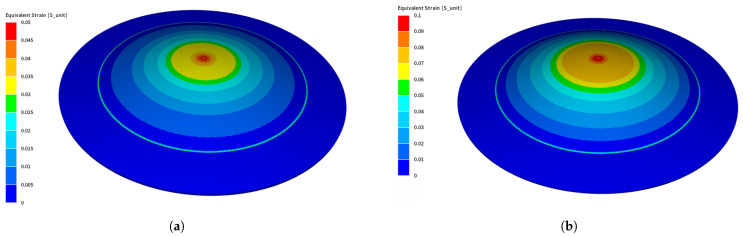
Equivalent strain of Steel and Al after indirect forming using MINI element. (**a**) 5kV; (**b**) 7kV.

The indirect forming process results will be the main focus of this section. More in-depth investigation will be tackled in this subsection. Figure 13 shows the equivalent strain of the workpiece in the indirect forming case, either for 5 kV or 7 kV. The equivalent strain in the indirect forming is more homogeneous than that shown in the direct forming case due to using the Aluminum workpiece underneath the Steel workpiece to enhance the forming process.

Likewise, numerical simulations are carried out for the indirect case using two elements: MINI and SHB. More deformation has been noticed for the Steel when used with Al since it makes use of the electrical conductivity of Al to gain more force to shape the Steel.

**Figure 14:**
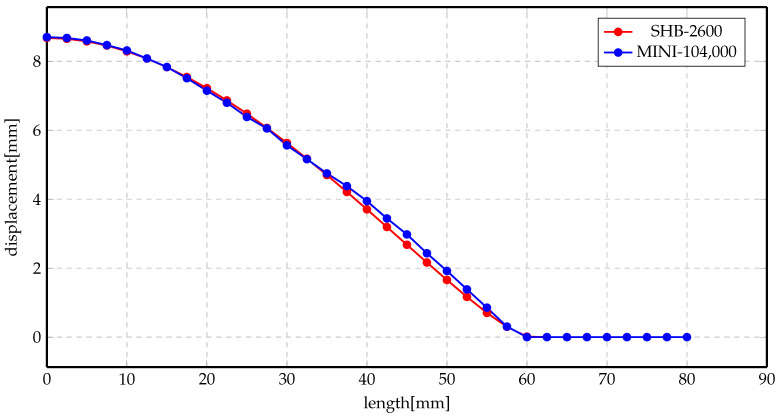
Displacement profile of indirect forming process at 5 kV using different elements.

**Figure 15:**
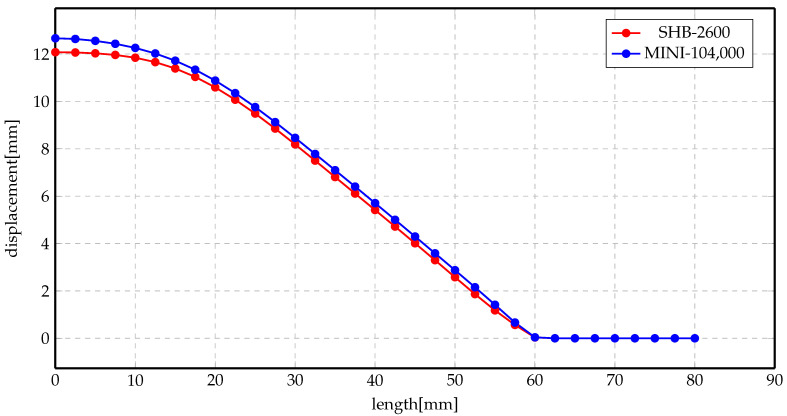
Displacement profile of indirect forming process at 7 kV using different elements.

Figure 14 and Figure 15 show the displacement profiles for SHB and MINI elements. The overall conclusion of these results is that the numerical results for both elements are very close even though the mesh sizes are different.

Overall, the results of the recently implemented element SHB showed very good agreement with its counterpart MINI element. These results are very encouraging to use this element in such complicated simulations as it proved its precision and efficiency. Although, more investigation is required to better compare the two elements, which will be introduced in the following section.

### 3.4. Simulation Time

**Figure 16:**
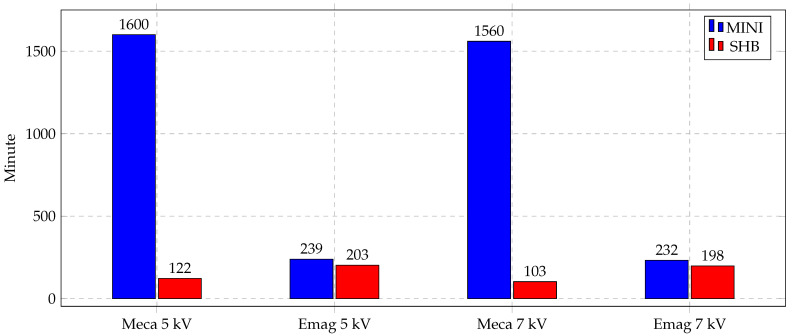
Simulation time of indirect forming process.

The aim of implementing the new element SHB is to use a special element for bending-dominated problems and obtain accurate results with a low number of elements. The usage of this element was very remarkable as the simulation time was greatly affected. Figure 16 shows a bar chart for the CPU time needed for both of the electromagnetic and mechanical simulations, separately, for indirect forming cases at two different voltages, 5 kV and 7 kV. In contrast to electromagnetic simulation times that were almost identical, the mechanical simulations times were greatly decreased using the SHB element. The simulation time is reduced by almost 10× in the SHB case.

**Figure 17:**
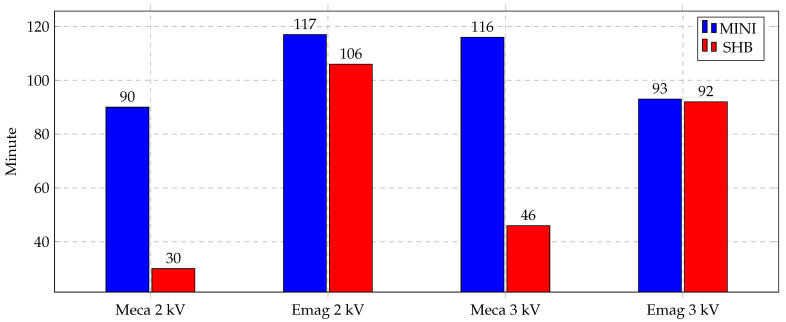
Simulation time of direct forming process of Al.

Similarly, the CPU time of the direct forming process represents the same trend. Figure 17 shows the bar chart of the direct forming of Al at two different voltages, 2 kV and 3 kV. Electromagnetic simulation times are almost identical, whereas the mechanical simulation time is much lower, 3× for the SHB element than the MINI element. The MINI element is used with a mesh of 52,000 tetra elements and SHB elements with 1300 elements. This difference in the number of elements causes the simulation time difference highlighted in these figures.

Finding a way to reduce the simulation time of the magnetic pulse forming is tremendously important, especially in the optimization processes. Optimization iterations have to be run to optimize the shape of the workpiece, study the effect of the workpiece’s thickness and carry out the material parameter identification process. This is considered a crucial milestone in the simulation of MPF processes and similar problems.

## 4. Discussion

This section is dedicated to giving a better insight of the results, along with discussing other results that explain the physical intuition of the process. Nevertheless, some challenges have been encountered while simulating the problem. Therefore, some of these problems are mentioned in the following subsections along with the proposed solutions. These problems include determining the final forming time, remeshing of the electromagnetic domain mesh and the effect of the mechanical mesh on the results. In the following subsections, the energy notion will be used to explain some of the difficulties that have been encountered while solving the problem. Therefore, it is important to explain the energy components that exist in this problem.

The initial energy input in the electromagnetic system is as follows:

(31) $$E_{in}=\frac{1}{2}\cdot C_{ele}\cdot V^{2}$$

where $E_{in}$ is the input energy, $C_{ele}$ is the electric capacitance of the coil and V is the voltage applied to the coil.

This energy is equal to the total energy that exists in the system:

(32) $$\begin{array}{cc} E_{total} & =E_{elec}+E_{therm}+E_{Meca}\\ E_{elec} & =0\\ E_{therm} & =0 \end{array}$$

In our case, we are not considering the $E_{elec}$ that is the dissipated energy due to electric resistance of the coil. Moreover, $E_{therm}$ is neglected, which is the dissipated thermal energy in the coil and in the mechanical system. This leaves only $E_{Meca}$ that is represented by:

(33) $$\begin{array}{cc} E_{Meca} & =E_{el}+E_{pl}+E_{kin}\\ E_{el} & =\int \sigma :{\dot{\varepsilon }}^{\mathrm{el}}dt\\ E_{pl} & =\int \sigma :{\dot{\varepsilon }}^{\mathrm{pl}}dt\\ E_{kin} & =\frac{1}{2}\rho \cdot v\left(x,t\right):v\left(x,t\right) \end{array}$$

where $E_{el},E_{pl},E_{kin}$ are the elastic strain energy, plastic strain energy and kinetic energy, respectively.

### 4.1. Final Forming Time

**Figure 18:**
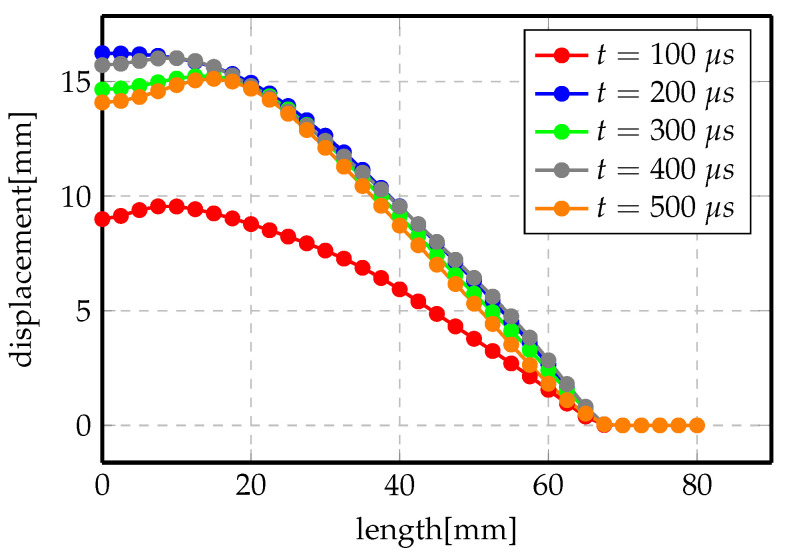
Displacement profile of direct forming process of Al at 3 kV.

One of the main issues with all these models is determining the final forming time. At the beginning, the simulation time was set to 150μs. However, the exact termination time of the process could not be determined and, by setting a too small value, we obtained an intermediate displacement profile, not the final one. Figure 18 shows a set of transient displacement profiles at different time steps. Therefore, a second stop condition has been added. It is induced from the calculation of a variation of the total energy of the system between the two last time steps. From this point of view, it is possible to set a threshold value at which calculation stops once reached. Otherwise, the recorded deformation will be a transient state and will not represent the real solution.

**Figure 19:**
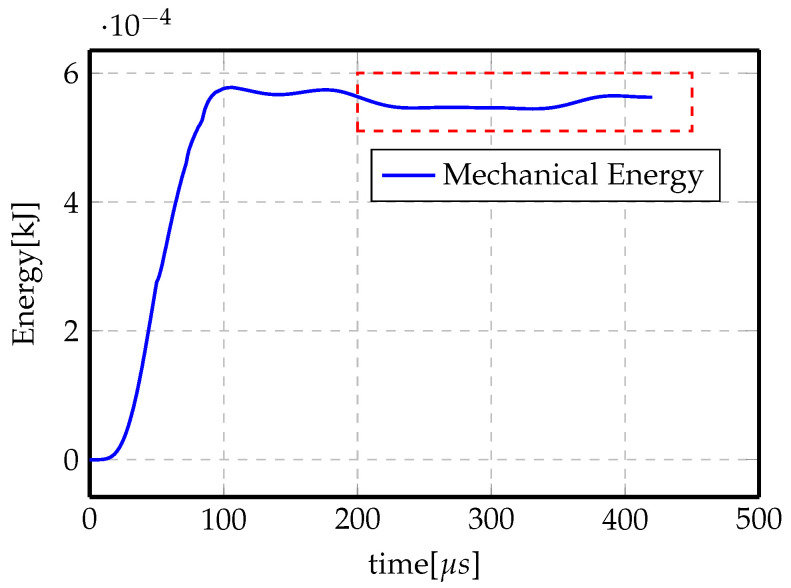
Total mechanical energy evolution with forming time.

To better understand this, Figure 19 shows the evolution of the mechanical energy (strain energy and kinetic energy) of the system in the indirect forming problem. The total energy increases at the beginning of the process in which the electromagnetic energy is maximum (electromagnetic energy represents the total energy in the system, it is then transformed to mechanical energy due to Lorentz force and thermal energy in the system and some loss as electrical energy in the coil). After some time, the system stops acquiring energy from the electromagnetic system and mechanical energy stays steady for the rest of the simulation time (highlighted in red dashed rectangle). The final simulation time is taken once the steady state value has been reached.

### 4.2. Remeshing of Electromagnetic Domain Mesh

**Figure 20:**
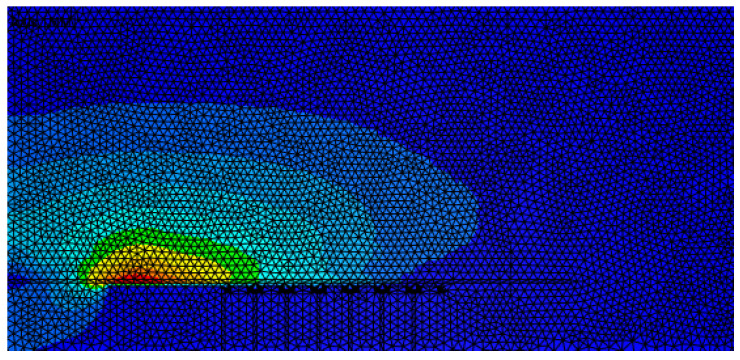
Electromagnetic mesh without remeshing.

**Figure 21:**
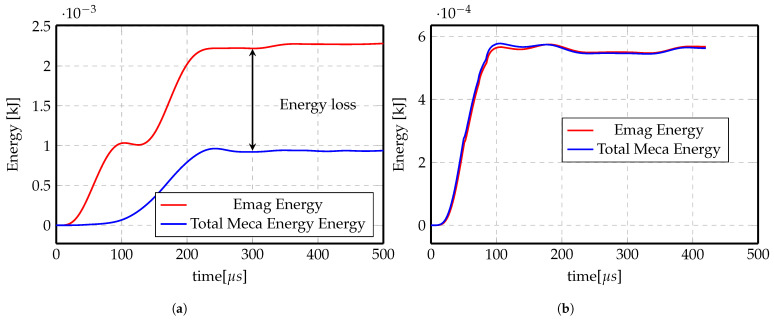
Electromagnetic energy vs. total mechanical energy of Al direct forming at 3 kV. (**a**) No Remeshing. (**b**) Remeshing.

At the beginning, the numerical simulations were carried out using a fixed but fine mesh in the electromagnetic domain, as shown in Figure 20, since it is more convenient and more computationally efficient as remeshing takes more computation time. Although the results were not very satisfactory, by checking the energy transferred between the electromagnetic mesh and the mechanical mesh shown in Figure 21a, it is obvious that at the end, there is energy loss.

**Figure 22:**
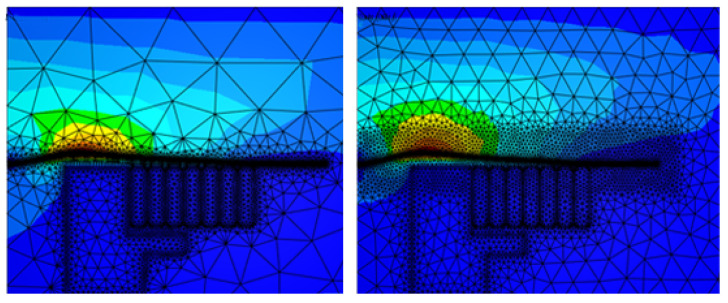
Electromagnetic mesh with remeshing.

On the other hand, Figure 21b shows the energy transferred with activating remeshing and the energy loss is negligible. The remeshing algorithm checks the deformation of the workpiece in the mechanical simulation and remeshes the surrounding of the workpiece in the electromagnetic simulation. Once the elements around the workpiece are highly deformed, the remesher refines these elements to maintain good mesh quality. This technique ensures that the mesh around the workpiece is always fine and clean, and thus guarantees correct calculations of the electromagnetic field and Lorentz forces, preventing the loss of energy. Figure 22 shows the electromagnetic mesh with activating remeshing before and after remeshing process.

Therefore, all the results in the previous subsections are obtained with remeshing activated. Although this increased the total simulation time, the obtained results can be trusted and a negligible amount of energy is lost.

### 4.3. Effect of Mechanical Mesh Refinement

**Figure 23:**
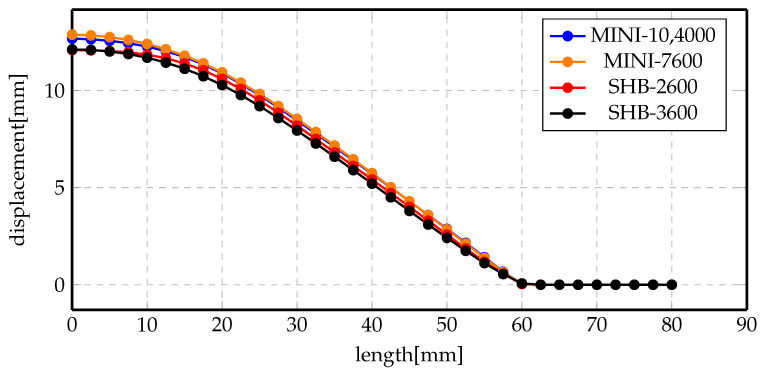
Displacement profile of indirect forming process at 7 kV using different elements and mesh sizes.

One of the questions that was intriguing while studying this process was the effect of mechanical mesh on the accuracy of the results. Thus, the indirect forming process has been simulated again with coarser mesh of the MINI element and its results have been compared to both the MINI fine mesh and SHB element. Figure 23 shows the displacement profile of the final deformed profiles for the previously mentioned elements and mesh sizes. The overall observation of the results is that displacement profiles are too close to be distinguishable. These results are not very comprehensible, since the MINI element should be stiffer than the SHB element and using a less number of elements should alter the displacement results. Moreover, in order to make sure that we have converged to mesh independent results, the simulation was repeated using more SHB elements, ≈3600 elements, as shown in the black curve. The difference between the red and the black curves are really small, meaning that the results obtained with the lower mesh size can be trusted.

**Figure 24:**
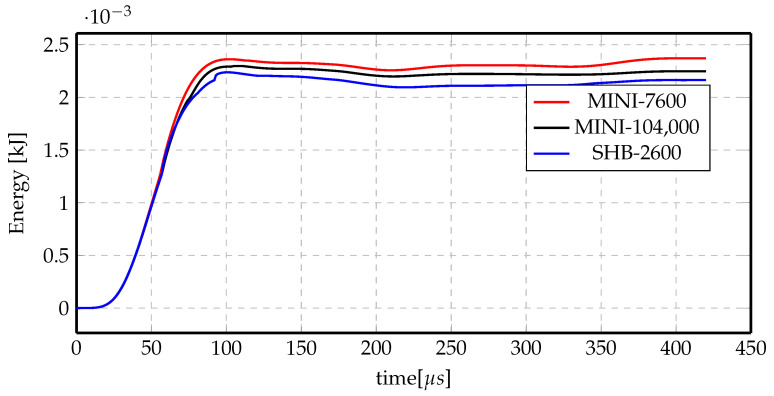
Total mechanical energy comparisons for different elements and mesh sizes for indirect forming at 7 kV.

However, more insight has been given to the mechanical energy of the three cases. Figure 24 shows the total mechanical energy for the three mesh cases. It is fairly noticeable that the energy of the SHB coarse mesh is the lowest and the MINI element with fine mesh energy is approaching it with minute difference. However, the energy of the MINI coarse mesh has a quite higher value. This means that given almost the same final deformation profile, the energy required is smallest in the case of the SHB element, even with coarse mesh, while it takes higher energy to achieve the same displacement in the case of using the MINI element with coarse mesh, and the energy decreases approaching the energy of the SHB element by refining the mesh. This clearly indicates that the MINI element is stiffer than the SHB element and would need much finer mesh to achieve comparable results to the SHB element.

## 5. Conclusions

An efficient approach for the simulation of the magnetic pulse forming process of thin sheet metals was developed by combining an electromagnetic solver, relying on Maxwell’s equations, with a mechanical solver, based on the conservation of momentum equations. In-depth analysis of the types of the magnetic pulse forming processes was carried out, namely on direct forming and indirect forming. Tetrahedral element (MINI) and solid-shell prism element were employed to solve the mechanical problem and quantitative comparisons were carried out to assess their performance in such applications. The overall results showed that the accuracy obtained with a low resolution SHB approach (low number of elements) was comparable to that of a high resolution MINI element based technique (high number of elements). The SHB element was shown to be less stiff than the MINI element. Finally, a computational cost study was carried out and demonstrated a higher computational efficiency for the SHB element since a smaller number of elements could be used while maintaining comparable accuracy to that of the MINI element. These results are very promising to study the performance of the SHB element, not only in MPF application but also in other applications. Many challenges were encountered during the simulation of this multi-physics problem and some solutions to overcome them were devised. First, the final forming time. It was very challenging to determine the exact final simulation time since the deformation takes place in the order of magnitude of milliseconds. Consequently, criteria based on measuring the change in the mechanical energy variation were adopted to find the termination time at which the energy variation is minimum. Second, the electromagnetic mesh had to be adjusted to consider the new mechanical deformation since the electromagnetic solution is highly dependent on the spatio-temporal evolution of the deformation of the workpiece in the mechanical problem. Hence, a remeshing strategy was utilized for the electromagnetic mesh and the results were compared to those conducted without the remeshing step.

## Figures and Tables

**Table 1:** Properties of the electromagnetic simulation.

Property	Value
Electrical resistivity of Al ($\rho_{Al}$)	4μΩ.cm
Electrical resistivity of Steel ($\rho_{Steel}$)	73μΩ.cm
Relative magnetic permeability of Al ($\mu_{Al}$)	1
Relative magnetic permeability of Steel ($\mu_{Steel}$)	1
Magnetic permeability in vacuum ($\mu_{0}$)	$4\;\pi .10^{-7}H.m^{-1}$
Machine parameters	$U_{\max}=7\;\mathrm{kV}$
	$R_{m}=1\;m\Omega$; $L_{m}=3.2\;\mu H$; $C_{m}=552\;\mu F$
Time step	1μs

**Table 2:** Material properties and Johnson–Cook law parameters of Al and Steel.

Property	Al	Steel
Elastic modulus (E)	73.1GPa	200GPa
Poisson ratio (ν)	0.279	0.3
A	83MPa	610MPa
B	426MPa	750MPa
C	0.025	0.008
n	0.35	0.25

**Table 3:** Boundary conditions for the indirect forming simulation.

	Al	Steel
Steel	Sliding	
3D manipulator (matrix)	Bilateral sticking	Bilateral sticking
